# Optimized fast GPU implementation of robust artificial-neural-networks for k-space interpolation (RAKI) reconstruction

**DOI:** 10.1371/journal.pone.0223315

**Published:** 2019-10-23

**Authors:** Chi Zhang, Seyed Amir Hossein Hosseini, Sebastian Weingärtner, Kâmil Uǧurbil, Steen Moeller, Mehmet Akçakaya

**Affiliations:** 1 Electrical and Computer Engineering, University of Minnesota, Minneapolis, MN, United States of America; 2 Center for Magnetic Resonance Research, University of Minnesota, Minneapolis, MN, United States of America; 3 Department of Imaging Physics, Delft University of Technology, Delft, Netherlands; University of Central Florida (UCF), UNITED STATES

## Abstract

**Background:**

Robust Artificial-neural-networks for k-space Interpolation (RAKI) is a recently proposed deep-learning-based reconstruction algorithm for parallel imaging. Its main premise is to perform k-space interpolation using convolutional neural networks (CNNs) trained on subject-specific autocalibration signal (ACS) data. Since training is performed individually for each subject, the reconstruction time is longer than approaches that pre-train on databases. In this study, we sought to reduce the computational time of RAKI.

**Methods:**

RAKI was implemented using CPU multi-processing and process pooling to maximize the utility of GPU resources. We also proposed an alternative CNN architecture that interpolates all output channels jointly for specific skipped k-space lines. This new architecture was compared to the original CNN architecture in RAKI, as well as to GRAPPA in phantom, brain and knee MRI datasets, both qualitatively and quantitatively.

**Results:**

The optimized GPU implementations were approximately 2-to-5-fold faster than a simple GPU implementation. The new CNN architecture further improved the computational time by 4-to-5-fold compared to the optimized GPU implementation using the original RAKI CNN architecture. It also provided significant improvement over GRAPPA both visually and quantitatively, although it performed slightly worse than the original RAKI CNN architecture.

**Conclusions:**

The proposed implementations of RAKI bring the computational time towards clinically acceptable ranges. The new CNN architecture yields faster training, albeit at a slight performance loss, which may be acceptable for faster visualization in some settings.

## Introduction

Long acquisition times remain a major drawback in MRI, creating a strong need for scan time acceleration. Parallel imaging is the most commonly used acceleration strategy in the clinic, where the local sensitivities of receiver coils are used for reconstruction [1–3]. One of the most utilized parallel imaging approaches is generalized autocalibrating partially parallel acquisition (GRAPPA), which estimates shift-invariant convolutional kernels from autocalibration signal (ACS) data to interpolate missing k-space lines from acquired ones [3].

Recently, there has been an interest in using machine learning techniques for accelerating MRI. These methods aim to generate more advanced regularizers by training on large amounts of datasets, with highly promising initial results [4–17]. Training in this setting requires large databases of MR images, and these methods do not exhibit any adaptation in a patient or scan-specific manner. An alternative recently proposed strategy, called robust artificial-neural-networks for k-space interpolation (RAKI) uses machine learning in a scan-specific manner, without the need for training databases [18]. RAKI interpolates missing k-space lines from acquired ones using several convolutional neural networks (CNNs) trained on subject-specific ACS data. The use of CNNs in RAKI was shown to improve the reconstruction quality over GRAPPA at high acceleration rates both visually and quantitatively [18].

In the original implementation of RAKI, CNNs were trained using a gradient descent approach with momentum [19] and central processing unit (CPU) processing. However, training multiple CNNs for each subject in this manner is a time-consuming task, leading to total reconstruction times of up to an hour, hindering its translational utility.

In this study, we sought to speed up RAKI reconstruction towards clinically acceptable computational times. We used a graphical processing unit (GPU) with CPU multi-processing to maximize the number of simultaneous training tasks, and proposed an alternative CNN architecture to reduce the number of required CNNs in the reconstruction and improve memory efficiency. Performance of different computational acceleration strategies and their combinations were compared in terms of run-time and reconstruction quality, using high-resolution phantom, brain and knee data.

## Materials and methods

### Overview of RAKI reconstruction

RAKI non-linearly estimates the missing k-space lines in a uniformly undersampled acquisition based on the acquired data, using multiple CNNs consisting of convolutional and non-linear activation layers. The reconstruction is similar to GRAPPA, but uses CNNs instead of linear convolutional kernels for interpolation in k-space [18]. For processing, the complex k-space is mapped to the real field, leading to a total of 2*n*_*c*_ input channels, where *n*_*c*_ is the number of coils. Let *S*(*k*_*x*_, *k*_*y*_, *j*) denote the k-space point (*k*_*x*_, *k*_*y*_) of the *j*^th^ channel. In RAKI, the unacquired lines are approximated by:

$$\begin{array}{l} {\left\{S(k_{x},k_{y}-m\Delta k_{y},j)\right\}}_{m\in \{1,2\ldots R-1\}}\\ \;\approx f_{j}\left({\left\{S(k_{x}-b_{x}\Delta k_{x}{,k}_{y}-Rb_{y}\Delta k_{y},1:2n_{c})\right\}}_{b_{x}\in [-B_{x},B_{x}],b_{y}\in [-B_{y},B_{y}]}\right) \end{array}$$ (1)

where Δ*k*_*x*_ and Δ*k*_*y*_ are the sampling intervals in frequency and phase encoding directions, *R* is the acceleration rate, *m* specifies an unacquired k-space position between two acquired lines, *B*_*x*_ and *B*_*y*_ are set by the size of the convolutional kernel along *k*_*x*_ and *k*_*y*_ directions, *f*_*j*_ represents the set of functions that estimate unacquired lines from acquired data, and 1: 2*n*_*c*_ denotes indexing across all channels. In RAKI, *f*_*j*_ is implemented using a three-layer CNN with the following structure [18]:

$$f(s)=w_{3}*ReLU(w_{2}*ReLU(w_{1}*s))$$ (2)

where * denotes convolution; ***w***_**1**_, ***w***_**2**_, ***w***_**3**_ are linear convolution kernels, of sizes $b_{1}^{x}\times b_{1}^{y}\times 2n_{c}\times n_{1},\;b_{2}^{x}\times b_{2}^{y}\times n_{1}\times n_{2}$, and $b_{3}^{x}\times b_{3}^{y}\times n_{2}\times (R‐1)$, respectively, and *ReLU*(*x*) = max(*x*,0). Thus, each CNN has (*R*—1) outputs, corresponding to the missing lines between uniformly undersampled k-space lines for a given channel. This approach necessitates a total of 2*n*_*c*_ CNNs [18]. In the learning phase of the algorithm, the convolutional kernels ***w***_**1**_, ***w***_**2**_, ***w***_**3**_ are estimated by minimizing mean square error loss function over the ACS region.

### GPU implementation using parallel multi-channel processing

RAKI was implemented on GPU using Tensorflow [20]. For optimizer, the gradient descent with momentum used in [18] utilizes a fixed gradient step, which leads to slow convergence [21]. Thus, Adaptive Moment Estimation (Adam) [22], which controls learning rates of all parameters by an exponential moving average window, as well as the first and second moments of historical gradients, was utilized in this study. This approach will be referred to as the naïve GPU implementation [21].

Further optimization of the GPU implementation was achieved as follows. Since RAKI trains 2*n*_*c*_ CNNs during a single reconstruction, where *n*_*c*_ is typically 30 or 32, and these CNNs are designed in a very compact structure that consist of only three convolutional layers and two activations, each individual training task in RAKI requires only limited GPU resources. Additionally, the subject-specific ACS data is comparably small compared to memory resources. Thus, since the training across channels is performed independently, multiple training tasks were parallelized to increase GPU utilization, and to provide speed up compared to sequential training procedures. For full GPU utilization, multiple CPU processes were launched simultaneously with each process allocating an individual training task on the GPU. For the CNN parameters used in this study, up to 16–20 CPU processes were concurrently executed to maintain peak GPU utilization. This CPU multi-processing allowed the GPU to commence processing of multiple calls at the same time. Furthermore, process pooling was utilized to avoid GPU overloading, while optimizing GPU resource usage.

### Line-by-Line CNN architecture for improved memory utilization

In the implementation in [18], each CNN estimated all the unacquired lines at a given coil, which will be referred to as coil-by-coil (CBC) architecture. Consequently, 2*n*_*c*_ CNNs needed to be trained during reconstruction. However, since training tasks are independent, each training task requires CPU-GPU communication proportional to the number of training tasks. Furthermore, distributing GPU resources into a high number of tasks, for instance 2*n*_*c*_, reduces available resources for each training task, leading to performance decrease. Therefore, in this study, we investigated an alternative architecture that improves the GPU memory usage. This architecture, which will be referred to as line-by-line (LBL), utilizes non-linear interpolation with CNNs, but each CNN estimates the unacquired lines for all channels for a given missing position *m*, as follows:

$$\begin{array}{l} S(k_{x},k_{y}-m\Delta k_{y},1:2n_{c})\\ \;\approx {f_{m}(S(k_{x}-b_{x}\Delta k_{x},k_{y}-Rb_{y}\Delta k_{y},1:2n_{c}))}_{b_{x}\in [-B_{x},B_{x}],b_{y}\in [-B_{y},B_{y}]} \end{array}$$ (3)

where 1:2*n*_*c*_ denotes indexing across all channels. Note the unacquired data are estimated by a CNN indexed by *m*, which outputs estimates in position *m* for all channels. Hence, this architecture reduces the CNN amount from 2*n*_*c*_ to *R*– 1. For instance, for *R* = 5, and *n*_*c*_ = 32, this leads to a 16-fold reduction. Note the kernel size of the third layer has been correspondingly changed to $b_{3}^{x}\times b_{3}^{y}\times n_{2}\times 2n_{c}$ for these CNNs, while the parameters of the other layers were kept fixed to maintain a fair comparison between the two architectures. The main advantage of this architecture from a computational perspective is the reduction of the number of CNNs that are used in reconstruction, which in turn reduces the data transfer between CPU and GPU, while allowing more GPU resources to be assigned to each training task.

### Implementation details

GPU-accelerated RAKI reconstruction was implemented using Tensorflow 1.7.0 and python 3.6.2, supported by CUDA 8.0 and CuDNN 7.0.5, on Linux kernel 3.10.0. The Python environment was created under Anaconda 5.1.0. All programs were run on a server with two Intel E5-2643 CPUs (6 cores each, 3.7 GHz), 256 GB memory and an NVIDIA Tesla V100 GPU (32 GB memory) with single precision. CPU-based RAKI reconstruction was implemented using Matlab 2016a and MatConvNet, as described in [18]. The RAKI networks shared the following parameters $b_{1}^{x}=5,\;b_{1}^{y}=2$, *n*_*1*_ = 32; $b_{1}^{x}=1,\;b_{1}^{y}=1$, *n*_*2*_ = 8; $b_{3}^{x}=3,\;b_{3}^{y}=2$. Prior to training, complex k-space data were mapped into real field, and then scaled into the range of [0, 0.015]. Parameters of Adam optimizer were set as: *α* = 0.001, *β*_*1*_ = 0.9, *β*_*2*_ = 0.999, *ε* = 10^−8^. Maximum training epoch was been chosen as 1000, and the training will be stopped prior than it if the normalized change of loss within 100 epochs is less than 0.0001. The multi-channel reconstruction result was combined by root-of-sum-of-squares. As weights were randomly initiated in CNN training, which affected the total run time, each run was repeated 10 times, and the reconstruction times were reported as mean ± standard deviation. GRAPPA reconstruction with a 5×4 kernel was also implemented for comparison with RAKI reconstructions.

### Phantom imaging

Phantom imaging was performed on a 3T Siemens Magnetom Prisma (Siemens Healthcare, Erlangen, Germany) system using a 32-channel receiver head coil-array and a head-shaped resolution phantom. A 2D multi-slice spoiled gradient recalled echo (GRE) sequence with the following parameters was used: FOV = 220×220 mm^2^, in-plane resolution = 0.7×0.7 mm^2^, matrix size = 320×320, slice thickness = 4 mm, TR/TE = 500 ms/15 ms, flip angle = 70°, 27 slices, bandwidth = 360 Hz/pixel. Retrospective sub-sampling was performed at *R* = 3, 4, 5, 6 with an ACS region of 40 lines at the center. Normalized MSE (NMSE) with respect to the fully sampled data was used to compare the accelerated RAKI implementations.

### Brain imaging

Brain imaging was performed on the same 3T system and on a 7T Siemens Magnex Scientific (Siemens Healthcare, Erlangen, Germany) system using a 32-channel receiver head coil-array. The imaging protocols were approved by the University of Minnesota institutional review board, and written informed consent was obtained from all participants before each examination for this HIPAA-compliant study. For 3T imaging, a T_1_-weighted 3D-MPRAGE sequence was acquired in a healthy subject (male, 41 years) with the following parameters: FOV = 224×224×179 mm^3^, resolution = 0.7×0.7×0.7 mm^3^, matrix size = 320×320, TR/TE = 2400 ms/2.2 ms, flip angle = 8°, bandwidth = 210 Hz/pixel, inversion time = 1000 ms, ACS lines = 40, with iPAT = 2 and 5. Furthermore, the *R* = 2 acquisition was also retrospectively undersampled to *R* = 4 and 6. For 7T imaging, 3D-MPRAGE was acquired in a healthy volunteer (male, 43 years) with the following parameters: FOV = 230×230×154 mm^3^, resolution = 0.6×0.6×0.6 mm^3^, TR/TE = 3100 ms/3.5 ms, flip angle = 6°, bandwidth = 140 Hz/pixel, inversion time = 1500 ms, ACS lines = 40, with *R* = 3, 4, 5, 6. Additionally, two averages were acquired for *R* = 5 and 6 data to mitigate the SNR loss from undersampling [18]. The k-space data was inverse Fourier transformed along the slice direction for all datasets, and a central slice was processed. For these acquisitions, where a fully-sampled reference was not available, reconstruction quality was assessed qualitatively.

### Knee imaging

Knee MRI data were obtained from the NYU fastMRI initiative database [23]. Experiments were performed on proton density weighted images with fat suppression, which was acquired using a 15-channel knee coil. Scan parameters of these datasets are as follows: echo train length = 4, matrix size = 320 × 320, TR/TE = 2870ms/33ms, in-plane resolution = 0.5×0.5mm^2^, slice thickness = 3mm, 36 slices, no gap between slices. These fully-sampled datasets were retrospectively undersampled with *R* = 2, 3 and 4, and 40 lines in the center of k-space were used as ACS data. Taking advantage of the copious amounts of data in this database, reconstructions were performed on 190 randomly selected slices across different subjects. Structural similarity index (SSIM), as well as NMSE with respect to fully sampled data was used to quantitatively measure the reconstruction quality. SSIM and NMSE performance with respect to the fully-sampled data was statistically compared using the Wilcoxon signed rank test among the two GPU implementations and GRAPPA over all the 190 instance for each acceleration rate. A type-I error of 0.05 was used to consider statistical significance.

## Results and discussion

### Phantom imaging

**Fig 1:**
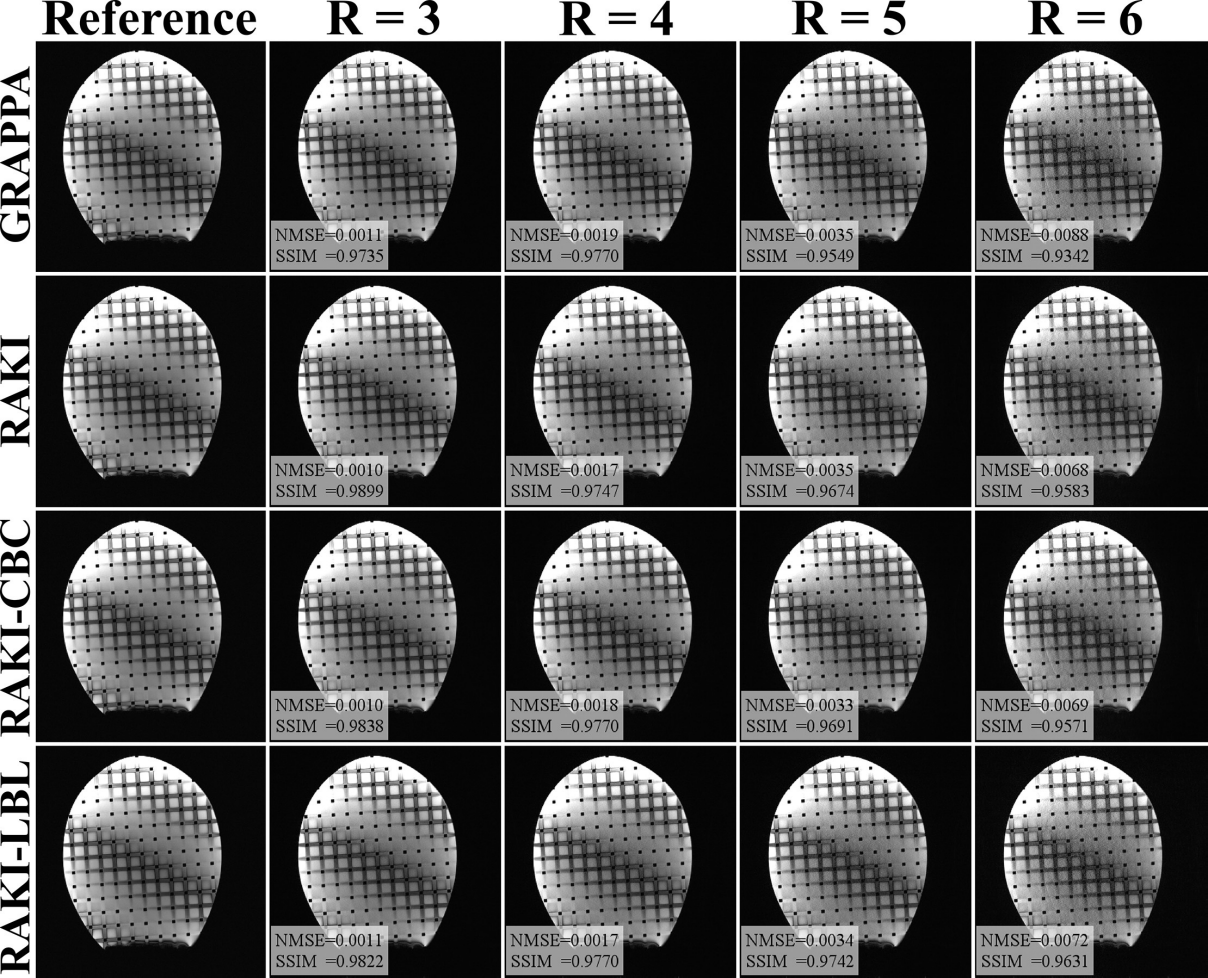
Reconstruction results of phantom imaging. Reconstruction was using the proposed GPU implementations with CPU multi-processing using the conventional coil-by-coil (CBC) and the novel line-by-line (LBL) architectures, and GRAPPA using 5 by 4 kernel for different acceleration rates. Different reconstructions for the same acceleration rate exhibit similar image quality and NMSE values. However, the optimized GPU-CBC strategy leads to 2.9 to 4.2-fold speed-ups compared to a naïve GPU implementation, while the optimized GPU-LBL strategy has further computational acceleration from 13.2 to 19.9-fold.

Reconstruction run times, including the learning phase, are listed in Table 1. Using the proposed GPU implementation with CPU multi-processing, 2.9 to 4.2-fold speed-up compared to naïve GPU implementation was achieved for different acceleration rates, with a maximum of 4.2-fold speed-up obtained for *R* = 3. Additional speed-up was achieved with the proposed LBL strategy, resulting in a 13.2 to 19.9-fold acceleration, where the maximum speed-up was again achieved for *R* = 3. Fig 1 shows the reconstruction results using GRAPPA, as well as the proposed RAKI GPU implementations with both CBC and LBL CNN architectures for different rates. The LBL GPU implementation uses a different architecture, but leads to virtually identical image quality for the phantom, while providing approximately 5-fold speed-up in computational time over the CBC implementation. This visual assessment is consistent with the NMSE values for this slice, 0.0010, 0.0018, 0.0033, 0.0069 for the RAKI GPU implementation with CBC architecture at *R* = 3 to 6 respectively, and NMSEs of 0.0011, 0.0017, 0.0034, 0.0072 for the LBL architecture for *R* = 3 to 6 respectively. Both CBC and LBL RAKI showed advantage over GRAPPA reconstruction, which had NMSEs of 0.0011, 0.0019, 0.0035, 0.0088 for *R* = 3 to 6, respectively.

**Table 1:** Run–times of all RAKI implementations.

	*R*	CPU-CBC (s)	Naïve GPU (s)	GPU-CBC (s)	Speed-up	GPU-LBL (s)	Speed-up
**Phantom** **(Fig 1)**							
	**3**	**8198 ± 43.8**	**159.4 ± 1.3**	**37.7 ± 0.2**	**4.2**	**8.0 ± 0.1**	**19.9**
	**4**	**7711 ± 14.4**	**155.2 ± 6.1**	**42.0 ± 0.4**	**3.7**	**9.1 ± 0.1**	**17.1**
	**5**	**6931 ± 19.2**	**147.1 ± 6.2**	**47.5 ± 0.3**	**3.1**	**10.9 ± 0.2**	**13.5**
	**6**	**5900 ± 30.6**	**158.4± 2.7**	**54.3 ± 1.1**	**2.9**	**12.1 ± 0.0**	**13.2**
**Brain 3T** **(Fig 2)**	**2**	**7583 ± 12.0**	**155.3 ± 2.3**	**32.8 ± 0.2**	**4.9**	**6.9 ± 0.1**	**22.2**
	**4**	**7589 ± 13.8**	**155.7 ± 2.2**	**40.4 ± 0.2**	**3.9**	**9.5 ± 0.1**	**16.4**
	**5**	**6840 ± 19.2**	**157.3 ± 2.4**	**46.0 ± 0.6**	**3.4**	**11.0 ± 0.2**	**14.3**
	**6**	**6055 ± 11.4**	**154.8 ± 1.6**	**51.8 ± 0.5**	**3.0**	**12.6 ± 0.4**	**12.3**
							
**Brain 7T** **(Fig 3)**	**3**	**9079 ± 13.2**	**157.7 ± 2.1**	**66.0 ± 3.8**	**2.4**	**8.5 ± 0.0**	**18.6**
	**4**	**8929 ± 12.6**	**168.6 ± 2.2**	**73.4 ± 5.6**	**2.3**	**10.1 ± 0.1**	**16.7**
	**5**	**8027 ± 13.8**	**165.0 ± 1.9**	**67.2 ± 1.1**	**2.5**	**11.4 ± 0.3**	**14.5**
	**6**	**7413 ± 29.4**	**168.9 ± 2.2**	**74.9 ± 2.6**	**2.3**	**12.9 ± 0.1**	**13.1**
**Knee** **(Fig 4)**	**2**	**4595 ± 27.6**	**80.3 ± 1.3**	**38.2 ± 1.9**	**2.1**	**6.9 ± 0.1**	**11.6**
	**3**	**4529 ± 24,4**	**80.5 ± 1.2**	**37.0 ± 0.6**	**2.2**	**7.7 ± 0.1**	**10.5**
	**4**	**4044 ± 34.8**	**78.8 ± 2.2**	**37.9 ± 0.8**	**2.1**	**9.2 ± 0.2**	**8.6**

Running times are reported in seconds. Means and standard deviations were calculated from 10 repetitions of the algorithm, with changes due to the random initialization of the weights in training. CBC and LBL refers to the output structure of the CNNs used in RAKI. The speed-ups in the table are reported with respect to the naïve GPU implementation.

### In-vivo imaging

Reconstruction run times for the different in vivo datasets, as well as for different *R* values are reported in Table 1. Similar to phantom imaging, 2.0 to 4.9 fold speed-ups with respect to naïve GPU implementations were achieved by using the proposed optimized GPU implementation over the in vivo datasets. Further speed-up from 8.6 to 22.2-fold is achieved by using the GPU implementation with the proposed LBL CNN architecture.

**Fig 2:**
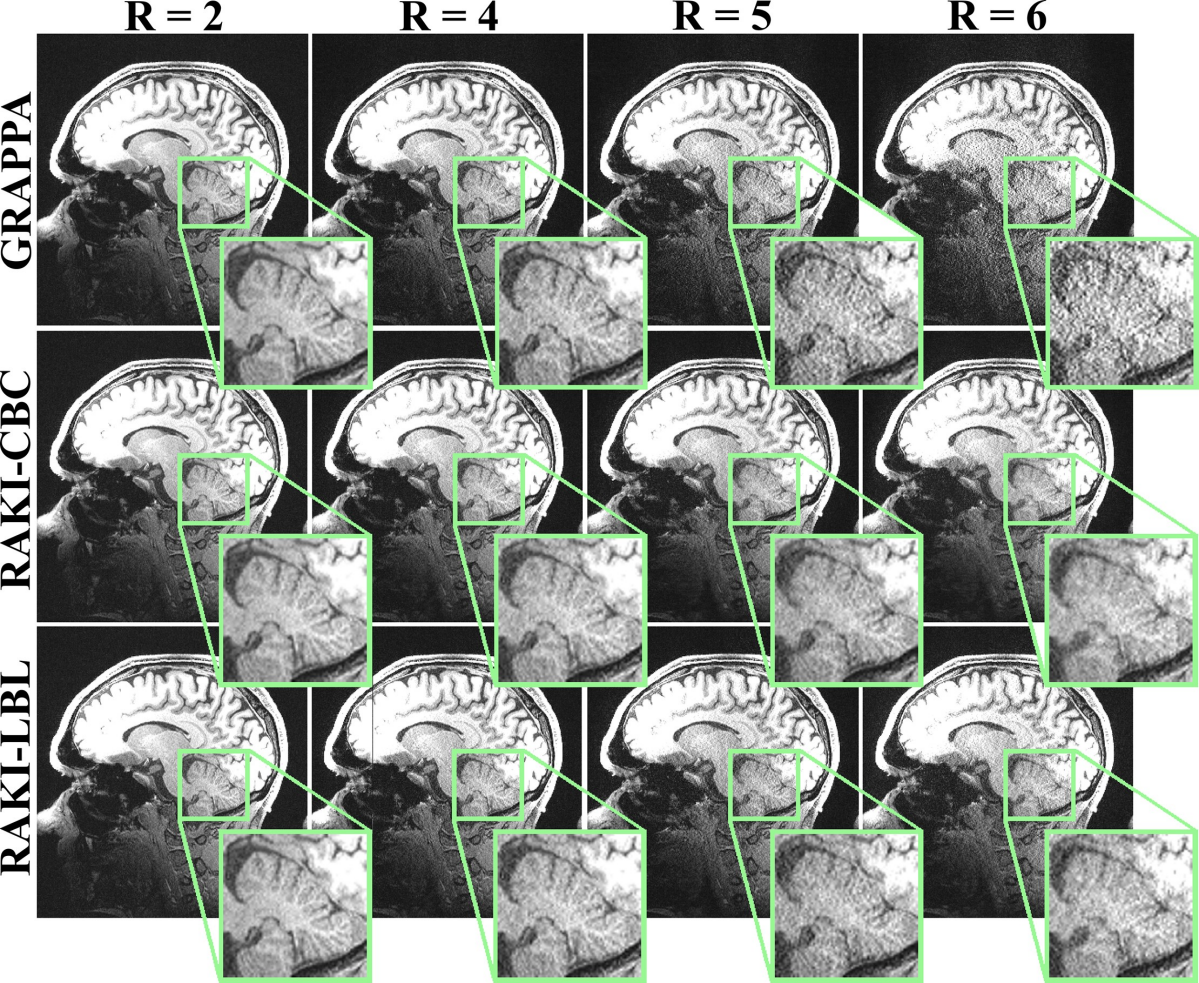
Reconstruction results of a central slice of MPRAGE data at 3T. The MPRAGE data was acquired at 3T with 0.7 mm isotropic resolution, using the proposed GPU implementations with CPU multi-processing using the conventional coil-by-coil (CBC) and the novel line-by-line (LBL) architectures for different acceleration rates. For *R* = 2 and 4, all reconstructions are visibly similar, but compared to a naïve GPU implementation, the proposed GPU strategies lead to computational speed-ups of up to 4.9 and 22.2-fold using the CBC and LBL architectures, respectively. For *R* = 5 and 6, slight noise amplification is observed for the RAKI-LBL implementation compared to the RAKI-CBC implementation. However, RAKI-LBL is still advantageous compared to GRAPPA in terms of noise resilience. The proposed GPU implementations of RAKI-CBC and RAKI-LBL led to 3.4 and 14.3-fold speed-ups over the naïve GPU implementation for these acceleration rates, respectively.

Fig 2 depicts the reconstruction results for a slice of the high-resolution 3T MPRAGE acquisition. There is a minor increase in noise amplification with the proposed fast GPU RAKI implementation with the LBL architecture as compared to the conventional CBC architecture at *R* = 5 and 6, while there are no visible differences for *R* = 2, 4. However, LBL RAKI still holds an advantage over GRAPPA in terms of visual quality and noise amplification, especially for *R* = 5, 6. Furthermore, the use of LBL architecture enabled computational speed-ups of 4.1 to 4.5-fold with respect to CBC architecture.

**Fig 3:**
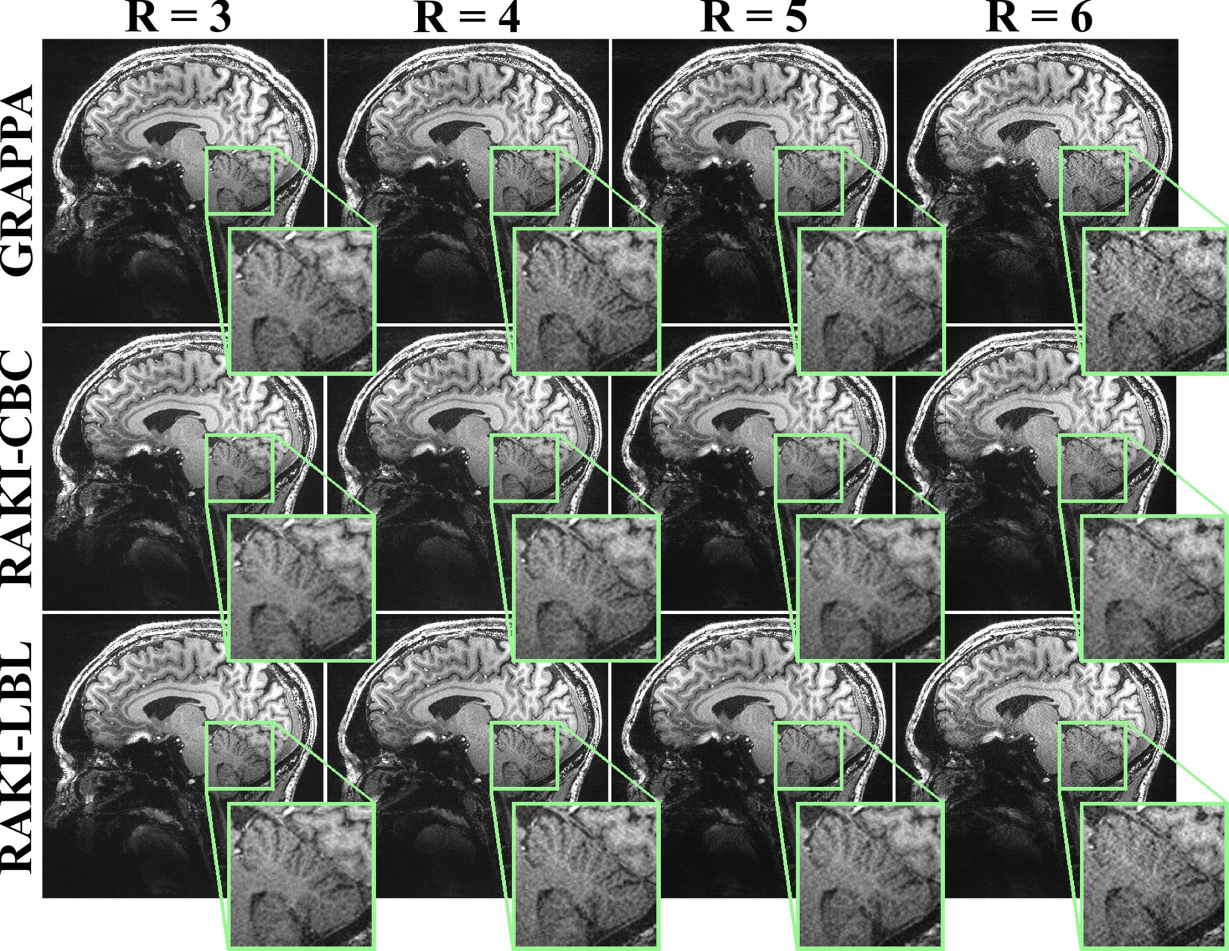
Reconstruction results of a central slice of MPRAGE data at 7T. The MPRAGE data was acquired at 7T with 0.6 mm isotropic resolution, using the proposed GPU implementations with CPU multi-processing using the conventional coil-by-coil (CBC) and the novel line-by-line (LBL) architectures for different acceleration rates. *R* = 5 and 6 data were acquired with two averages for reduced SNR penalty. For *R* = 3 and 4, reconstructions are visibly similar. Compared to the naïve GPU implementation, with the GPU strategies leading to computational speed-ups of up to 2.4 and 18.6-fold using the CBC and LBL architectures, respectively. Slight noise amplification with the RAKI-LBL approach over the RAKI-CBC approaches are visible for *R* = 5 and 6. However, RAKI-LBL offers better noise resilience over GRAPPA. GPU implementation of RAKI-CBC and RAKI-LBL led to 2.5 and 14.5-fold computational speed-ups over the naïve GPU implementation for these rates, respectively.

Fig 3 depicts a reconstructed slice for 7T MPRAGE acquisition at 0.6mm isotropic resolution. Similar reconstruction characteristics are observed in this scenario as well. Minor noise amplification is observed with the proposed fast GPU RAKI implementation with the LBL architecture compared to the CBC architecture, but only at the higher acceleration rates of 5 and 6. Up to approximately 8-fold acceleration is achieved with the LBL GPU implementation, when compared to the CBC GPU approach for this dataset. RAKI reconstructions with both CBC and LBL architectures show better noise resilience over GRAPPA.

**Fig 4:**
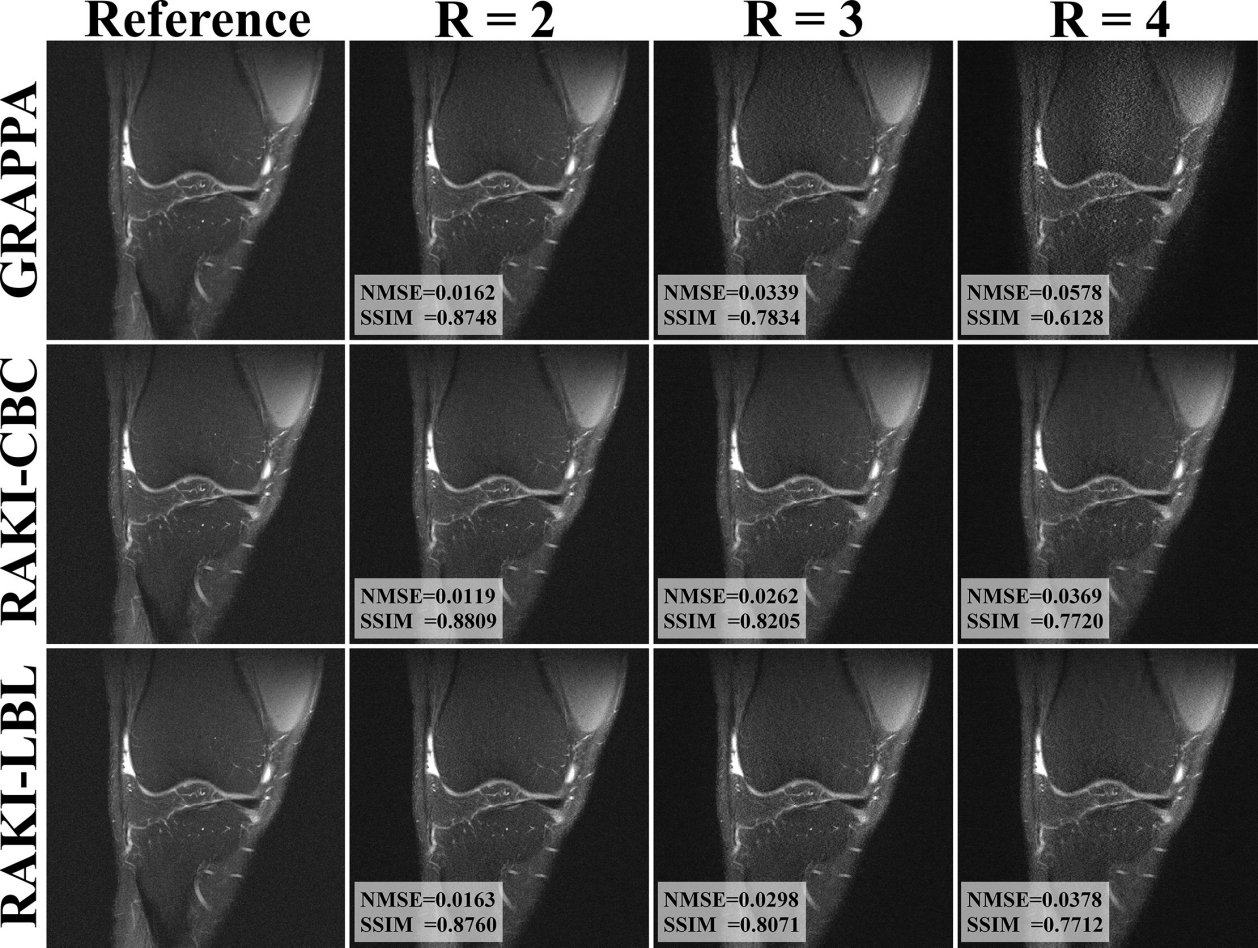
Reconstruction results of a proton density weighted knee image with fat suppression. Fully sampled data was provided by fastMRI dataset [23]. Reconstructions using GRAPPA, and proposed GPU implementations CBC and LBL are shown. For *R* = 2 case there is no visible difference between reconstruction results. For *R* = 3, RAKI shows advantages in noise resilience compared to GRAPPA. Both CBC and LBL architectures lead to less noise than GRAPPA. This advantage is even more apparent at *R* = 4, where RAKI reconstructions show considerably lower noise level than GRAPPA. For both *R* = 3 and 4 cases, RAKI-CBC and RAKI-LBL have no substantial visual difference. Quantitative SSIM and NMSE metrics confirm these observations.

Fig 4 displays reconstructions of proton density weighted knee images with fat suppression from the fastMRI dataset [23]. For *R* = 2, no visual differences are observed among the three reconstruction methods, which is consistent with SSIM values of 0.8543, 0.8643 and 0.8584, for GRAPPA, RAKI GPU-CBC and RAKI GPU-LBL respectively. For *R* = 3, both CBC and LBL RAKI show advantage over GRAPPA in terms of reconstruction noise visually, while CBC and LBL RAKI methods are visually similar. The SSIM values for GRAPPA, CBC and LBL are 0.7373, 0.7988 and 0.7807 respectively, consistent with visual observations. For *R* = 4, GRAPPA suffers from even higher reconstruction noise, while RAKI offers higher reconstruction fidelity for both CBC and LBL implementations, with minor improvements with CBC over LBL. The SSIM values are 0.5955, 0.7534 and 0.7382 for GRAPPA, CBC and LBL respectively.

**Fig 5:**
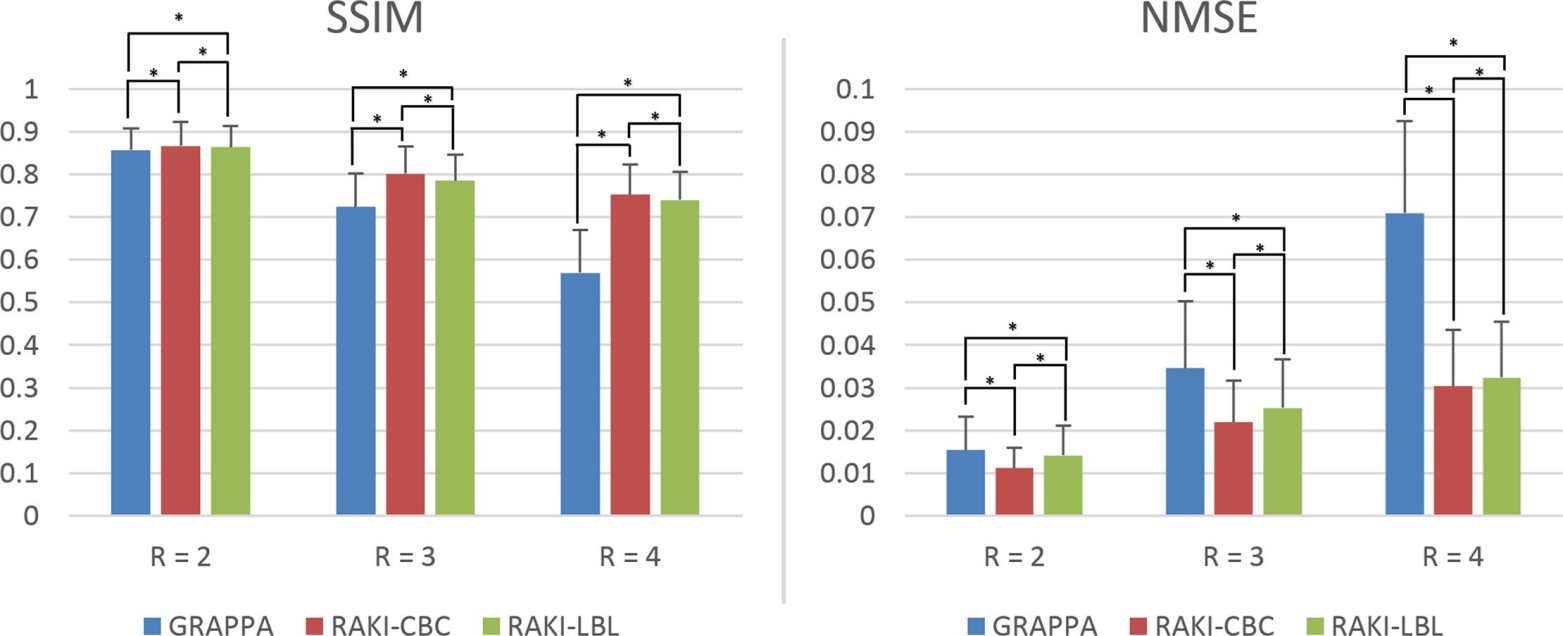
Mean structural similarity index (SSIM) and normalized mean squared error (NMSE) for different methods. SSIM and NMSE of GRAPPA, RAKI-CBC and RAKI-LBL for 190 proton density weighted knee data with fat suppression from the fastMRI dataset [23]. Error bars represent standard deviation across datasets. SSIM results showing both RAKI-CBC and RAKI-LBL offers better image quality than GRAPPA, with 24.0% improvement at *R* = 4. Similar observations apply to NMSE. All differences between methods and across rates were statistically significant (*P* < 0.05), which are marked with *.

Fig 5 summarizes the mean and standard deviation of the SSIM and NMSE metrics for GRAPPA, and CBC and LBL RAKI over the 190 knee MRI datasets from the fastMRI database [23]. CBC RAKI performs best at all rates, while LBL RAKI also outperforms GRAPPA at all rates, with 0.5%, 5.9% and 24.0% SSIM improvement at *R* = 2, 3 and 4. The relative differences between CBC RAKI and LBL RAKI were smaller for SSIM at 0.7%, 2.3% and 2.0% at *R* = 2, 3 and 4. Similar observations are made for the NMSE metric, where LBL RAKI outperforms GRAPPA by 7.8%, 26.9% and 54.3% at *R* = 2, 3, 4, while the relative difference between CBC RAKI and GRAPPA is 27.3%, 36.7% and 57.1% at *R* = 2, 3, 4. All the differences for SSIM and NMSE were statistically significant at all rates (*P* < 0.05).

## Discussion

In this study, we proposed various approaches to accelerate RAKI reconstruction. Individual CNN training was accelerated by GPU-aided implementation. Multiple CNNs for RAKI reconstruction were trained in a parallel manner based on CPU multi-processing and process pooling techniques, in order to maximize GPU utilization and achieve further acceleration. Additionally, an LBL CNN architecture for RAKI was proposed to reduce the number of CNNs required for reconstruction, which afforded additional speed-up with no significant changes in image quality. These efforts reduced RAKI run-time from hour-long CPU processing towards clinically acceptable range of seconds.

Acceleration of deep learning techniques using massive parallelization is an active area of research. To date, most studies focused on the case where one large training task is performed at a time [24, 25]. The computational acceleration need in RAKI is different since multiple compact CNNs are trained independently. Due to the comparably small size of the individual CNNs, powerful GPUs are not at full use if these networks are trained subsequently. Hence, our approach was to parallelize the training on a single GPU without compromising the performance of each individual training. This strategy of allocating multiple training tasks on a single GPU facilitated peak performance resulting in faster RAKI reconstructions.

Further computational speed-up was achieved by reducing the number of CNNs required for a RAKI reconstruction. Conventional RAKI requires 2*n*_*c*_ CNNs, where each CNN corresponds to a certain coil over the real field. In this work, we proposed an LBL network structure that outputs reconstructions across all coils for a specific missing k-space line, in order to reduce CNN requirement in RAKI reconstruction. This strategy significantly reduced the number of CNNs that needed to be trained, further improving the reconstruction times.

Two different GPU implementations were investigated in this study. The first one utilized the same CBC structure as in [18], but used GPU and CPU multi-processing. Compared to a naïve GPU implementation, using this strategy improved processing speed from several minutes to less than a minute. Additionally, the use of fixed learning rate in the original CPU implementation was identified as a limitation [18], which was ameliorated in this study by using a more advanced optimization approach [21, 22]. Overall, our strategies reduced the hour-long CPU run-time in [18] to seconds, while providing the best reconstruction quality, robustness, even for high acceleration rates The LBL strategy gave further considerable speed-up, with similar reconstruction quality at moderate acceleration rates of up to 4, although consistent but minor noise amplification was observed for high-resolution brain imaging at high acceleration rates of 5 and 6, while the visual differences were not substantial for the knee datasets. This indicates a trade-off between reconstruction quality and speed-up, which may be acceptable in certain settings, considering the additional 5 to 8-fold speed-up in computational time with this approach.

The two CNN architectures considered in this study had the same number of layers, kernel sizes, and number of outputs except for the last layer. This led to different numbers of parameters that needed to be learnt. For the CBC architecture, the number of parameters is given as 640*n*_*c*_ + 208 + 48*R* for each CNN. 2*n*_*c*_ such CNNs resulting $1280n_{c}^{2}$ + 516*n*_*c*_ + 96*Rn*_*c*_ parameters in total, whereas for the LBL architecture, there were 736*n*_*c*_ + 208 parameters for each CNN, and totally (736*n*_*c*_ + 208)(*R*—1) parameters for the (*R*—1) CNNs. Note in this study, *n*_*c*_ *=* 32 for phantom and brain imaging, and *n*_*c*_ = 15 for knee imaging. Thus for *R* ≤ 6, the CBC architecture had more than 4-fold as many as parameters as the LBL. This suggests that the LBL architecture can potentially support deeper CNNs with more outputs per layer. However, for a fair comparison between the two architectures, while avoiding any additional confounding factors, both architectures were tested with the same number of layers and other network parameters in this study. According to our experiments, using larger kernel sizes did not improve the reconstruction quality for either GRAPPA or RAKI.

Further acceleration may be achieved by using multi-task learning. In multi-task learning [26–28], a single network offers multiple output utilities, by allowing partial parameter sharing between different output branches. Aided by this mechanism, reconstruction of the whole multi-coil image may be accomplished using a single multi-task network, rather than multiple individual networks. This strategy facilitates overlaps between the network architectures for multiple outputs. Thus, it has the potential to provide a more efficient reconstruction procedure than existing RAKI-CBC and RAKI-LBL. Since the scope of this study is to accelerate RAKI reconstruction proposed in [18], we have tried to keep algorithmic modification to a minimum. However, future studies using more advanced multi-task learning models to further accelerate the reconstruction are warranted.

For the GPU implementation, there is a non-trivial overhead due to data transfer to GPU, which impacts the overall run-time. To quantify the effect of this overhead, we computed a data transfer to computation ratio for the different implementations. For RAKI-CBC, this ratio was between 0.4 and 0.6, while for RAKI-LBL, the ratio was between 2.2 to 2.4. Thus, for the latter implementation more than half of the total run-time is spent on data transfer to the GPU. Further reduction in this overhead would be beneficial for the implementations, but are currently unavoidable due to hardware limitations.

While RAKI enables scan-specific machine learning reconstruction, more conventional machine learning reconstruction algorithms have also been considered in the literature. These methods require large databases of fully-sampled images for training. Transfer learning methods have also been proposed to partially address the need for large databases, which may not be available in all target applications. In transfer learning, neural networks are pre-trained on an available large database, and then fine-tuned on smaller datasets for the specific application [29, 30]. However, these methods still require fully-sampled data for training. Thus, they may not be applicable to scenarios, where it is infeasible to acquire such datasets, for instance for the high-resolution whole-brain imaging considered in this paper, since the scan time would be prohibitive. Additionally, the databases used for training with or without transfer learning may have limitations on pathologies of interest, bringing risks in generalizability for diagnosis of rare pathologies [31]. This latter problem is also addressed by the scan-specific nature of RAKI.

In summary, we proposed several strategies to accelerate RAKI reconstructions in order to facilitate translation of this scan-specific machine learning parallel imaging reconstruction to the clinic. The original CBC RAKI reconstruction was accelerated by a factor of 2.1 to 4.9 compared to a naïve GPU implementation. Additional speed-up of up 8.6 to 22.2-fold compared to a naïve GPU implementation, was achieved using a novel LBL CNN structure in RAKI, further bringing the computational time towards clinically acceptable range.
